# Novel insights into voltage-gated ion channels: Translational breakthroughs in medical oncology

**DOI:** 10.1080/19336950.2023.2297605

**Published:** 2023-12-28

**Authors:** Minas Sakellakis, Sung Mi Yoon, Jashan Reet, Athanasios Chalkias

**Affiliations:** a Department of Medicine, Jacobi North Central Bronx Hospital, Bronx, USA; b Institute for Translational Medicine and Therapeutics, University of Pennsylvania Perelman School of Medicine, Philadelphia, PA, USA; c Outcomes Research Consortium, Cleveland, OH, USA

**Keywords:** Ion, channels, regeneration, cancer, treatment, translational research

## Abstract

Preclinical evidence suggests that voltage gradients can act as a kind of top-down master regulator during embryogenesis and orchestrate downstream molecular-genetic pathways during organ regeneration or repair. Moreover, electrical stimulation shifts response to injury toward regeneration instead of healing or scarring. Cancer and embryogenesis not only share common phenotypical features but also commonly upregulated molecular pathways. Voltage-gated ion channel activity is directly or indirectly linked to the pathogenesis of cancer hallmarks, while experimental and clinical studies suggest that their modulation, e.g., by anesthetic agents, may exert antitumor effects. A large recent clinical trial served as a proof-of-principle for the benefit of preoperative use of topical sodium channel blockade as a potential anticancer strategy against early human breast cancers. Regardless of whether ion channel aberrations are primary or secondary cancer drivers, understanding the functional consequences of these events may guide us toward the development of novel therapeutic approaches.

## Introduction

Although genetic information is responsible for the synthesis of proteins during human and animal embryogenesis, it remains uncertain whether the instructions for protein–protein interactions during embryogenesis are also “hidden” in the genetic code. State-of-the-art research suggests that experimental modulation of voltage gradients induced by ion channels and pumps can orchestrate downstream molecular-genetic pathways of organ regeneration or repair, acting as a kind of top-down master regulator [1–3].

## Bioelectrical signals drive embryogenesis and regeneration

In experiments conducted in *planaria* worms, Levin et al. were able to show that a bioelectrical layer, rather than genetic information, was orchestrating the regenerative patterns in the worms after amputation of large portions of their body [1]. Scientists were able to grow two different heads instead of a head and a tail, simply by using appropriate bioelectrical signals [1]. Other studies also showcased that electrical stimulation shifts the response to injury toward regeneration instead of healing or scarring. Herrera-Rincon et al. reported that the use of a bioreactor device at amputated sites in adult African aquatic clawed frogs (*Xenopus laevis)* triggered a degree of regenerative response that is normally not seen [1–3].

Transcriptome analysis and RNA sequencing revealed that the bioelectrical signals altered gene expression patterns in cells at the amputation site. Genes involved in scar-tissue formation signaling and immune response were downregulated, while genes associated with oxidative stress, white blood cell activity, or serotonergic signaling were upregulated. Compared to control frogs, the ones with the device developed thicker bones, with more prominent vascularization and innervation, while their swimming patterns were closer to that of the non-amputated frogs [3].

It has also been suggested that ectopic organ formation can be triggered via an appropriate manipulation of voltage gradients [4]. Moreover, experiments showed that a multi-component sleeve assembly that encompassed the amputated site was effective in supporting the early stages in murine fingertip regeneration, when combined with electrical stimulation [5]. Several cell types exhibit galvanotaxis, while in early vertebrate embryos, electric fields not only regulate cell polarization but can also provide important cues during cellular movement and pattern formation [6]. Experiments in zebrafish suggested that a mutation in potassium channels that affects pore formation can alter the migration of melanosomes. Altering bioelectrical events during early embryogenesis in *Xaenopus* tadpoles may also cause melanocytes to inappropriately colonize organs or tissues [6].

In vitro studies have shown that electrical stimulation can induce cell migration, while in vivo studies suggest that osteogenesis, vasculogenesis, extracellular matrix deposition, and cell proliferation can all be increased by appropriate electric stimulation [4]. These results add to the growing body of evidence suggesting that tiny bioelectrical signals can surge among and through the cells and regulate gene expression to promote organogenesis and tissue or organ regeneration. These bioelectrical signals are the results of ion channel-induced cell polarity and voltage gradient changes [4].

Ion channels are not only involved in cellular electrogenesis and excitability but they can also form macromolecular complexes and interact with signaling molecules or adhesion proteins. In addition, they regulate cellular proliferation, differentiation, apoptosis, as well as cellular metabolism. Changes in the ion composition inside the cells affect several cellular events and molecular pathways. One notable example is cell movement which requires an ion channel-orchestrated sequence of cellular protrusions and retractions [7].

## Effects on cancer

Cancer is characterized by uncontrolled cellular proliferation, along with increased and inappropriate migration, apoptosis evasion, and abnormal neo-angiogenesis. Cancer and embryogenesis not only share common phenotypical features but also commonly upregulated molecular pathways (Table 1) [8–18]. Given the similarities, it is reasonable to hypothesize that constantly altered or defective bioelectrical signaling triggered by ion channel aberrations may be a key driver in cancer development and progression. Genomic defects usually associated with cancer, sometimes do not accurately predict tumor aggressiveness, pointing toward the existence of additional drivers [19]. There is a growing body of evidence in the literature suggesting a pro-tumorigenic effect of various ion channel aberrations [7]. Mutations or expression losses in voltage-gated ion channel genes, as well as abnormal expression/function are linked to several tumor types. Table 2 provides a summary of the roles that voltage-gated ion channels play in the development of cancer (Table 2) [20–34]. In breast, prostate, cervical, and ovarian cancers, preclinical evidence suggests that voltage-gated sodium channel alterations directly affect cell cycle regulation, tumor cell movement, metastasis, and calcium levels within cells [21–25]. Voltage-gated potassium channels have been implicated in apoptosis induction, cell movement, and cell growth regulation in tumors such as melanoma, glioblastoma, breast, and prostate cancer [21,25–29]. Overwhelming evidence also supports the critical role of voltage-gated calcium channels in various stages of development and progression of prostate cancer, melanoma, glioblastoma, and adrenal adenomas. They are known to regulate tumor cell growth rate, apoptosis induction, and metastasis in bone tissue [21,30–32]. Voltage-gated chloride channels impact prostate cancer and gliomas by regulating parameters such as tumor cell growth rate, cell movement, volume level maintenance, and calcium homeostasis inside the cells [33,34]. These findings emphasize the role played by various voltage-gated ion channels in several types of cancers.

**Table 1.** Common pathways that play a role in embryogenesis and cancer [8–18]. CNS=central nervous system, SCLC=small-cell lung cancer, ALL=acute lymphoblastic leukemia.

Pathway	Role in Embryogenesis	Role in Cancer
Wnt	Involved in myogenesis, CNS, tooth development and limb patterning	Wnt alterations affect beta-catenin signaling and Cyclin D1 activity
Hedgehog	Involved in development of CNS, lung, gut, face, limbs, blood, testis, etc	Aberrations involved in pathogenesis of medulloblastoma, SCLC
Notch	Controls embryonic and postnatal tissue differentiation, cell fate specification and stem cell maintenance	Aberrations involved in neuroblastomas, SCLC, T-cell ALL, prostate, skin, and cervical cancer
Protease activated receptors	Anterior-posterior cell patterning, separation of eye fields	Potentially linked with colon, lung, laryngeal, renal and breast cancer
Bone morphogenetic peptide/TGF beta	Affects right-left symmetry, mesoderm formation, distal limb morphogenesis, endochondral bone formation	Implicated in colon, lung cancer, specific lymphomas. Likely plays a role in bone metastasis formation

**Table 2.** The role of ion channels and P-class pumps in malignancy [20–34].

Ion channel	Malignancies implicated	Effect
Voltage-gated sodium channels	Breast, prostate, cervical, ovarian	Cell cycle arrest, regulates tumor cell migration, metastasis, regulates intracellular calcium levels
Voltage-gated potassium channels	Melanoma, glioblastoma, prostate, breast cancer	Apoptosis induction, migration, mobility, regulates cell proliferation
Voltage-gated calcium channels	Prostate, melanoma, glioblastoma, adrenal adenomas	Regulates tumor cell proliferation, apoptosis, migration, bone metastasis
Voltage-gated chloride channels	Prostate, gliomas	Regulates tumor cell proliferation, migration, cell-volume regulation, intracellular calcium homeostasis

Ion channel activity is directly or indirectly involved in the pathogenesis of all cancer hallmarks, while several experimental and clinical studies suggest that their modulation may exert antitumor effects [35]. A notable example is the positive association between anesthetic drug use and increased overall survival in cancer patients. Potential mechanisms have been proposed, including innate and adaptive immune system modulation, or a direct effect on ion channel signaling [36]. Interestingly, local anesthetics continue to inhibit the activity and function of voltage-gated sodium channels (VGSCs) beyond the intraoperative period [37]. In a study using SW620 cell line (metastatic colon cancer), ropivacaine was found to act as a potent inhibitor of metastatic cancer cell invasion [38]. Ropivacaine was also shown to play a role in reducing prostate cancer metastatic potential, by altering intracellular ion concentration and cellular homeostasis [7]. Lidocaine and bupivacaine are other VGSC blockers which displayed antitumor effects in experimental models [7,39,40].

Non-anesthetic VGSC inhibitors, such as ranolazine or phenytoin, have also been shown to exert antitumor effects in preclinical models of breast cancer [41,42]. A recent retrospective study investigated the association between the use of VGSC inhibitors and cancer-specific mortality in patients with breast, prostate, and colorectal cancer [43]. Despite the limitations of retrospective design and the fact that additional confounding factors may underlie the associations, it was shown that VGSC-inhibiting antiarrhythmic exposure, but not anticonvulsant, was associated with improved cancer-specific mortality.

Accumulative evidence suggests that VGSC are aberrantly expressed in non-excitable cancer cells from different origins but not in cognate normal tissues. Some diseases with mutations in VGSC genes might predispose to tumorigenesis, which further suggests a causal association. Several tumors display abnormally high intracellular concentrations of sodium ions. Many critical cellular activities that are highly upregulated in cancer (including glutamine and glucose import) are dependent on the electrochemical Na^+^ gradient maintenance across the plasma membrane. Moreover, altered pH regulation and membrane potential depolarization have been proposed to play important roles during cancer metastasis. VGSCs also act as cell adhesion molecules [43–47]. Overall, these findings lead scientists to propose that abnormal expression of VGSCs may result in the re-expression of a fetal phenotype in a group of pathological cells.

On the other hand, the heterogeneous family of potassium channels constitute arguably the largest group of ion channels. Abnormal voltage-gated potassium channel expression is documented in numerous cancer types, while they constitute well-known molecular targets for the development of novel cancer therapies [48]. It has been suggested that their expression can modify multiple cancer progression mechanisms, including cell cycle control, proliferation, cell migration, invasion, and apoptosis [49]. For example, voltage-gated K^+^ channels Kv10.1 are restrictively expressed in the brain, but are also overexpressed in 70% of solid tumors of various origins [50]. In vitro and in vivo studies showed that Kv10.1 suppression via known Kv10.1 inhibitors, targeted monoclonal antibodies or using siRNA generated apoptosis and decreased cell proliferation and migration, while also sensitized tumor cells to antimetabolic agents [50–53].

Kv11.1 (or HERG) is another extensively studied voltage-gated K^+^ channel which is overexpressed in various cancer cell lines. It is physiologically expressed in cardiac myocytes, smooth muscles, neurons, and neuroendocrine cells. It has been suggested that its aberrant expression in cancer regulates proliferation, invasiveness, and migration. Although Kv11.1 blockers have shown antineoplastic effects in vivo, it comes with a caveat of QT prolongation and torsadogenesis that could lead to fatal arrhythmia. Thus, current studies and clinical trials focus on non-torsadogenic Kv11.1 blockers [54,55]. On the other hand, experiments suggested that HERG1 hyperstimulation may result in a senescence-induced irreversible breast cancer cell proliferation inhibition [56]. Although the findings regarding Kv11.1 channels are conflicting, they suggest an important role in cancer biology that needs to be further elucidated.

The role of voltage-gated calcium channels in the pathogenesis of different cancers has also been increasingly recognized. Extensive research is currently focused on their use as therapeutic targets or predictive markers against several tumor types. Calcium ion homeostasis disruption is a well-known phenomenon in cancer. There are several classes of calcium channels via which extracellular Ca^2+^ enter cells [7,57,58]. Intracellular Ca^2+^ influx is usually the result of calcium repletion in the endoplasmic reticulum, which subsequently repletes the stores and activates several downstream signaling pathways. Although not a requirement for cancer initiation by itself, the consequences of aberrant intracellular calcium concentration, calcium oscillations, and calcium-regulated signaling can be significant and contribute to cancer initiation and progression [7,57,58]. However, the association between calcium channel blocker use and prevalence of various cancers in retrospective studies have shown mixed results [58].

Voltage-gated chloride channels are transmembrane proteins that regulate chloride ion homeostasis in different cells. They play a role in a variety of physiological roles, such as volume homeostasis, regulation of excitable cells, cell cycle regulation, pH regulation, transepithelial transport, and organic solute transport. Cell volume alterations are pivotal for cellular proliferation and apoptosis [7,59–61]. Aberrations in cell volume regulation can predispose to apoptosis, while strengthening the regulatory response to volume decrease may confer resistance to apoptosis. Accumulative evidence suggests that upregulation of various voltage-gated chloride channels might be associated with the pathogenesis of different tumor types, hence they are currently being studied as promising antineoplastic targets [7,59–61].

## Evidence from clinical studies

A recent large prospective clinical study by Badwe et al. investigated the impact on survival of preoperative, peritumoral infiltration of lidocaine in patients with early breast cancer [62,63]. Early disease was defined as operable cancer with clinically negative or limited nodal disease and no evidence of distant metastasis. In this open-label, multicenter randomized study, 1583 patients who were not assigned to receive neoadjuvant chemotherapy received peritumoral injection of 0.5% lidocaine followed by surgery (786 patients) or surgery alone (797 patients). All patients received standard adjuvant postoperative treatments. After a median follow-up of 68 months, topical lidocaine increased 5-year disease-free survival (DFS) (hazard ratio [HR]: 0.74; 95% CI: 0.58 to 0.95; p-value = 0.017) and 5-year overall survival (OS) rates (HR: 0.71; 95% CI: 0.53 to 0.94; p-value = 0.019). Patients who received lidocaine had an almost 4% improvement in overall survival, which is comparable to the benefit received by other current standard-of-care adjuvant interventions. The effect of topical lidocaine was similar in all the examined subgroups defined by menopausal status, tumor size, nodal infiltration status, hormone receptor status, or human epidermal growth factor receptor 2 status. Moreover, the benefit was present regardless of whether the patients underwent mastectomy or removal of only the tumor and surrounding tissue. Interestingly, no adverse events accompanied lidocaine injection [62]. Limitations of the study include the single-nation nature, the lack of placebo control, and the fact that investigators were unblinded to the study's intervention. Despite the limitations, these results are remarkable, given the lack of toxicity, the ease and low cost of intervention, and the large sample size of the trial [62].

In addition, surgical resection of a tumor and the surgical stress response may predispose to metastasis [63]. Surgical excision can potentially modify immune function, activate neural and proinflammatory signaling, and may even induce dissemination of circulating tumor cells and increase prometastatic pathways [63]. The use of local anesthetic drugs exerts effects that can theoretically pose anticancer activity. For example, pain alleviation may decrease surgical stress response. Other studies have also linked lidocaine with the alteration of pathways critical for tumor cell proliferation, invasion, angiogenesis, and apoptosis evasion [63]. The study by Badwe et al. serves as a proof-of-principle for the role of voltage-gated sodium channel blockade as a potential anticancer strategy against human breast cancers. Apart from the obvious importance as a local treatment strategy, it calls for the planning of further carefully designed clinical trials to expand on the concept of voltage-gated ion channels as tumor drivers. Although local lidocaine may act more through affecting the microenvironment, a direct and long-lasting effect on tumor cells is also highly likely. This is of particular importance because we should always consider the possibility that cancer cells have already escaped to the systemic circulation at the time of surgery.

## Discussion

Voltage-gated ion channel activity is responsible for the production and transmission of signals that orchestrate critical steps in human and animal embryogenesis. Cancer-promoting aberrations in these channels may result in excessive activation of signals that favor cell proliferation, apoptosis evasion, cell migration, or neovascularization. It may also suppress growth-inhibitory signals [21]. Frequently, tissue and organ homeostasis is achieved via a cross-talk between activating and inhibitory signals. Defects in normal cellular cross-talk impairs tissue homeostasis, increasing the risk for abnormal events, such as the formation of a tumor. The latter is composed of a group of cells, which lack the ability to interact with the surrounding stroma in a way that promotes healthy homeostasis [64]. Abnormal bioelectrical signals might result in events with a degree of similarity to embryogenesis and development. However, embryogenesis is programmed to occur in the growing organism, under circumstances that are not present anymore in the adult organism. This provides a useful theoretical framework that is able to explain how simple changes in voltage-gated ion channel activity can contribute to tumorigenesis. Regardless if these aberrations are the true primary cancer drivers or the result of other drivers, understanding the functional consequences of these events may guide us toward the development of novel therapeutic approaches.

## Conclusion

There is a growing body of evidence suggesting that voltage-gated ion channels may act as tumor drivers in several cancer types. Given their importance in human physiology, the efforts to understand bioelectric signals and ion channel blockers should intensify.

## Data Availability

Data sharing is not applicable to this article because no new data was created or analyzed in this study.

## Highlights

- Cancer and embryogenesis not only share common phenotypical features but also commonly upregulated molecular pathways.
- Elegant experiments showed that bioelectrical signals act as a top-down master regulator during embryogenesis, while electrical signals shift response to injury toward regeneration instead of healing or scarring.
- Voltage-gated ion channel activity is directly or indirectly linked to the pathogenesis of all cancer hallmarks, while experimental and clinical studies suggest that their modulation may exert antitumor effects.
- A large recent clinical study showed that preoperative administration of local anesthetics in patients with early breast cancer can improve survival.
- The efforts to understand and therapeutically exploit bioelectric signals in cancer should intensify.
